# The Protective Effects of Ginseng Polysaccharides and Their Effective Subfraction against Dextran Sodium Sulfate-Induced Colitis

**DOI:** 10.3390/foods11060890

**Published:** 2022-03-21

**Authors:** Shanshan Li, Xiaohui Huo, Yuli Qi, Duoduo Ren, Zhiman Li, Di Qu, Yinshi Sun

**Affiliations:** 1 Institute of Special Animal and Plant Sciences, Chinese Academy of Agricultural Sciences, Changchun 130112, China; shanshali123456@163.com (S.L.); xiaohui.114@163.com (X.H.); qiyuli521@163.com (Y.Q.); renduoduo611@163.com (D.R.); lzm091215@163.com (Z.L.); qudi2022@163.com (D.Q.); 2 Department of Biology, College of Biological and Pharmaceutical Engineering, Jilin Agricultural Science and Technology University, Jilin 132101, China

**Keywords:** gut microbiota, colitis, *Panax ginseng* polysaccharides, pectin, short-chain fatty acids

## Abstract

Polysaccharides from *Panax ginseng* are natural carbohydrates with multiple activities. However, little was known about its functions on colitis. In this study, we aim to investigate the protective effects of ginseng polysaccharides and its effective subfraction on dextran sodium sulfate (DSS)-induced colitis. Water soluble ginseng polysaccharides (WGP) were obtained from dry ginseng root, then purified to neutral fraction (WGPN) and acidic fraction (WGPA) by ion exchange chromatography. An animal model was constructed with male Wistar rats, which were treated with a normal diet (con group), DSS (DSS group), WGP (WGP group), WGPN (WGPN group), and WGPA (WGPA group), respectively. Both WGP and WGPA alleviated the colitis symptoms and colon structure changes of colitis rats. They decreased the disease activity index (DAI) scores and improved colon health; reduced colon damage and recovered the intestinal barrier via regulating the tight-junction-related proteins (ZO-1 and Occludin); downregulated inflammatory cytokines (IL-1β, IL-2, IL-6, and IL-17) and inhibited the TLR4/MyD88/NF-κB-signaling pathway in the colon; regulated the diversity and composition of gut microbiota, especially the relative abundance of *Ruminococcus*; enhanced the production of SCFAs. In conclusion, WGP exerted a protective effect against colitis with its acidic fraction (WGPA) as an effective fraction. The results support the utilization and investigation of ginseng polysaccharides as a potential intervention strategy for the prevention of colitis.

## 1. Introduction

Colitis is a chronic gastrointestinal disease with a high incidence and an unclear pathogenesis [1]. Patients with colitis exhibit a series of inflammatory symptoms, such as diarrhea, vomiting, abdominal pain, rapid weight loss, and blood and/or pus in the stool. These symptoms easily affect the daily lives of patients. During the process of development of colitis, a significant shift in the nature of the gut microbiota is observed, i.e., an increase in pathogenic bacteria and a decrease in beneficial bacteria are detected [2,3]. Moreover, the gut microbiota regulates the gut microecological environment by acting on the production of short-chain fatty acids (SCFAs) and other metabolites, thus affecting the signaling pathways and immune cytokines of the host [4]. The balance between pro-inflammatory and anti-inflammatory factors is also important for the colitis status. Once this balance is destroyed in the gastrointestinal system, the intestinal mucosa is damaged and exhibits a disease status, which might also have adverse effects on the isolation of intestinal contents and nutrient absorption and may even endanger lives [5,6].

Polysaccharides, as bioactive macromolecules, are also reportedly beneficial to intestinal health. For example, *Astragalus* polysaccharides alleviate dextran sodium sulfate (DSS)-induced colitis by inhibiting the NF-κB activation pathway [7]. *Dictyophora indusiate* polysaccharide could alleviate the severity of colitis by improving the gut epithelial integrity and inflammatory reactions [8]. Polysaccharides extracted from *Blidingia minima* showed an anti-inflammatory effect on DSS-treated colitis by repairing colonic dysfunction and improving colonic morphology, infiltration, and the expression of tight junction, pro-inflammatory cytokines, as well as the protein levels of NF-κB in colonic tissue [9]. Polysaccharides also affect the diversity and composition of gut microbiota, such as polysaccharides from *Ganoderma lucidum* [10], and *Panax ginseng* [11] can alleviate the intestinal microbial environment in mice with different diseases, and *Dictyophora indusiata* polysaccharides [12] can promote recovery from antibiotic-driven intestinal dysbiosis and improve gut epithelial barrier function in mice. It also reported that polysaccharides derived from *Schisandra chinensis* [13] and *Astragalus membranaceus* [14] could improve the composition of gut microbiota. Different sources of polysaccharides showed various effects on gut microbiota. Therefore, plant polysaccharides might be a potential active ingredient in the treatment of colitis.

*Panax ginseng* has been listed as a health-care food in China since 2012. Polysaccharides are the most important active components of ginseng. Our previous studies showed that ginseng polysaccharides can regulate the composition and diversity of the gut microbiota and promote the recovery of the mucosa in mice with antibiotic-associated diarrhea [11]. It has also been reported that ginseng polysaccharides could improve the DSS-induced colitis by inhibiting the JAK2/STAT1/NLPR3 inflammasome-signaling pathway in mice [15], as well as enhance ginsenoside Rb1 absorption and affect gut microbial metabolism [16]. However, there are very few reports about the effective subfraction in ginseng polysaccharides on anti-colitis activity. Therefore, this study aimed to analyze the effects and potential mechanism of ginseng polysaccharides and its subfractions on anti-colitis activity to provide new approaches to the treatment and prevention of colitis.

## 2. Materials and Methods

### 2.1. Preparation of WGP, WGPN, and WGPA

The roots of *Panax ginseng* were purchased from Wanliang ginseng market in Fusong, Jilin, which was verified as four-year-old, field-grown *Panax ginseng* C.A. Meyer by Professor Wei Li from Jilin Agricultural University. The ginseng roots (500 g) were extracted in hot distilled water (5 L) three times, combined the filtration and concentrated to 1 L, then precipitated by anhydrous ethanol with a final concentration of 80%, and deproteinized in Sevag reagent (1 L) three times. Subsequently, the aqueous layer was freeze dried, and the total soluble polysaccharide extract (WGP) was obtained. Next, WGP was loaded onto a DEAE-Sepharose Fast Flow column (4 cm × 37 cm), eluted with distilled water and 0.5 mol L^−1^ NaCl, then dialyzed (0.5 mol L^−1^ NaCl fraction; Mw, 3500 Da) and freeze dried; the neutral fraction (WGPN, eluted with distilled water) and the acidic fraction (WGPA, eluted with 0.5 mol L^−1^ NaCl) were obtained [17,18]. The total carbohydrate and uronic acid content was determined according to methods previously reported [19]. Protein content was determined using the Dumas nitrogen analyzer (NDA 701). The monosaccharide compositions of the WGP, WGPN, and WGPA samples were analyzed by HPLC (Shimadzu, Kyoto, Japan) after acid hydrolysis and PMP derivatization [13].

### 2.2. Animal Groups and Experimental Design

Five-week-old male Wistar rats (weight, 160–180 g) were purchased from Changsheng Biotechnology Co., Ltd. (Liaoning, China), caged separately, and raised in a temperature- and humidity-controlled lab environment (22 ± 1 °C; relative humidity, 50 ± 5%) with a strict 12 h light/dark cycle. The rats were placed in the environment for three days before the experiment. The study protocol (No. TCS-2017019, January 2017) was reviewed and approved by the Laboratory Animal Management and Ethics Committee of the Institute of Special Animal and Plant Sciences, Chinese Academy of Agricultural Sciences. All animal experiments were performed in strict accordance with the Guidelines for the Care and Use of Laboratory Animals recommended by the Institute of Special Animal and Plant Sciences, Chinese Academy of Agricultural Sciences and the Chinese Legislation on Laboratory Animals. Every effort was made to maximize the well-being of rats and to minimize their suffering.

Forty rats were equally distributed into five groups (con, DSS, WGP, WGPN, and WGPA). The rats in the DSS, WGP, WGPN, and WGPA groups were administered 5% DSS (molecular weight, 36–50 kDa; MP Biomedical Solon, OH, USA) in the drinking water on a modeling period of seven days, whereas the rats in the con group were administered normal drinking water without DSS. After the modeling period, the rats in the WGP, WGPN, and WGPA groups received WGP (100 mg kg^−1^), WGPN (60 mg kg^−1^), and WGPA (35 mg kg^−1^), respectively, twice daily by gastric gavage during a recovery period of seven days. Dose of WGP were selected as per our previous study, whereas doses of WGPN and WGPA were determined by their yields from WGP, i.e., 61.8% and 26.5%, respectively. Concomitantly, the rats in the con and DSS groups received physiological saline.

During the experimental period, body weight was measured daily. The disease activity index (DAI) was used to assess the severity of colitis (Table 1) [20]. At the same time, we have refined the standard scores of stool consistency and stool occult blood. The DAI score of each rat was calculated as the average scores of the three indicators. Stool occult blood was tested with stool occult blood kit benzidine method (Leagene, TC0511).

### 2.3. Sample Collection

Fresh fecal samples were collected aseptically from rats on the eighth day of the recovery period. Subsequently, the rats were anesthetized with isoflurane. The lengths of the colons were measured. The bowel contents were rinsed with pre-cooled normal saline, and the colon tissue was collected, weighed, and segmented. The end segment of the colon tissue (~5–8 cm) was collected and divided into three parts in which the parts near the rectum were fixed in 10% neutral formalin solution for histological observation, whereas other parts were stored at −80 °C for use in mRNA and protein extraction.

### 2.4. Histological Assessment

The histological analysis was performed as previously reported [13].

### 2.5. Western Blotting

Proteins of colon samples (0.1 mg) were extracted using a protein extraction kit (No. C510003-0050, Sangon Biotech. Inc., Shanghai, China), and we followed the procedures according to the instructions. The protein content was determined using the BCA assay (No. C503021-0500, Sangon Biotech. Inc.). The buffer solution (0.1 M Tris–HCl, 20% glycerol, 4% SDS, and 0.01% bromophenol blue) was added to the protein lysates in a ratio of 1:4 and boiled for 10 min. Aliquots of protein were subjected to 10% SDS-PAGE. The separated proteins were transferred to a PVDF membrane for 1 h at 200 mA. The membrane was pre-blocked with 5% nonfat milk in TBST, with gentle shaking at 4 °C overnight. Each membrane was washed five times for 5 min and incubated with the secondary horseradish peroxidase-linked antibody. Quantitation of detected bands was performed with the Image Pro-Plus software. Each density was normalized using the intensity of the corresponding β-actin protein band as loading controls. The density of the control group for relative comparison was standardized as 1.0 to compare across groups or the ratio of aim protein/β-actin. Anti-IL-1β (A1112), anti-IL-2 (A16317), anti-IL-6 (A0286), anti-IL-17 (A10052), and anti-NF-κB (A19653) antibodies were purchased from ABclonal (Wuhan, China) Biotechnology Co., LTD. Anti-ZO-1 (ab221547), anti-occludin (ab216327), anti-TLR4 (ab22048), and anti-MyD88 (ab219413) antibodies were purchased from Abcam (Cambridge, MA, USA).

### 2.6. Gut Microbiota Analysis

Before further analysis, total bacterial genomic DNA was extracted from the rat fecal samples using a Fast DNA SPIN Extraction Kit (MP Biomedicals, Santa Ana, CA, USA), followed by quantification using a NanoDrop ND-1000 Spectrophotometer (Thermo Fisher Scientific, Inc., Waltham, MA, USA) and agarose gel electrophoresis. The V3–V4 region of the 16S rRNA gene was amplified by PCR using a forward primer (5′–ACTCCTACGGGAGGCAGCA–3′) and a reverse primer (5′–GGACTACHVGGGTWTCTAAT–3′). Moreover, a DNA library was constructed, optimized, and sequenced using the Illumina MiSeq System. The original data from high-throughput sequencing were screened and controlled according to the sequence quality using the QIIME software (version 1.8.0). Length distribution of the sequences contained in all samples was analyzed statistically using the R software package (v3.2.0). Sequences with similarity greater than 97% were defined as an operational taxonomic unit (OUT) using the online QIIME software, and the most abundant sequence within each OUT was selected as the representative sequence [21]. All representative reads were annotated and compared with the template sequence in the Greengenes database [22].

### 2.7. Contents of SCFAs

The SCFAs of each rat were analyzed as previously reported [13].

### 2.8. Statistical Analyses

Statistical analyses were performed using the GraphPad Prism software (version 5.01), and the significance of differences was analyzed using ANOVA (Tukey’s test). Significance was set at *p* < 0.05.

## 3. Results

### 3.1. Physiochemical Features of the Ginseng Polysaccharide Fractions

The water-soluble polysaccharide (WGP) from ginseng roots was obtained after water extraction, ethanol precipitation, and deproteination as a yield of 10.1%. Total carbohydrate contents, uronic acid contents, protein contents, and monosaccharide composition were analyzed and are shown in Table 2. WGP was composed of rhamnose (Rha, 2.7%), galacturonic acid (GalA, 25.7%), galactose (Gal, 15.1%), arabinose (Ara, 13.9%), and glucose (Glc, 42.6%). WGPN and WGPA were purified from WGP using DEAE-Sepharose Fastflow chromatography via elution with distilled water and 0.5 mol L^−1^ NaCl, respectively. WGPN was the neutral polysaccharide fraction and exhibited the main structure features of a starch-like glucan [11] composed of Gal, Ara, and Glc with a ratio of 3.3%, 1.4%, and 95.3%, respectively. WGPA was mainly composed of Rha (3.8%), GalA (44.2%), Gal (18.0%), Ara (15.4%), and Glc (13.6%). WGPA contains type I rhamnogalacturonan (RG-I) and homogalacturonan (HG) regions, which serve as acidic pectins that are rich in uronic acid [11,12].

### 3.2. WGP and Its Subfractions Alleviated DSS-Induced Colitis

**Figure 1:**
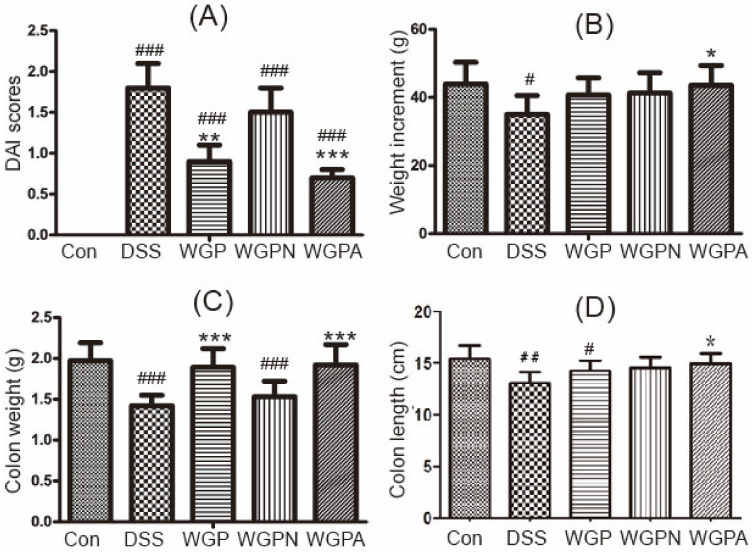
The influence of ginseng polysaccharide fractions on colon health. (**A**) DAI scores; (**B**) weight increment; (**C**) colon weight; (**D**) colon length. The bars represent the mean ± SD, *n* = 8; # *p* < 0.05, ## *p* < 0.01, ### *p* < 0.001 compared with the con group; * *p* < 0.05, ** *p* < 0.01, *** *p* < 0.001 compared with the DSS group. DAI, disease activity index; con, control group; DSS, colitis group; WGP, WGP treatment group; WGPN, WGPN treatment group; WGPA, WGPA treatment group.

Both WGP and WGPA decreased the DAIs significantly compared with the DSS group (Figure 1A), with WGPA exhibiting the lowest DAI value. Weight increment results showed that WGPA increased the body weight after the recovery period compared with DSS treatment (Figure 1B). Colon length and colon weight were significantly reduced in the DSS group compared with the con group (Figure 1C,D). Moreover, colon weight and length of the WGPA group were significantly different from that of the DSS group. These results suggested that WGP obviously alleviated the gut injury resulting from colitis, while WGPA was the effective subfraction.

### 3.3. Ginseng Polysaccharide Fractions Reduced Colon Damage and Recovered Intestinal Barrier

**Figure 2:**
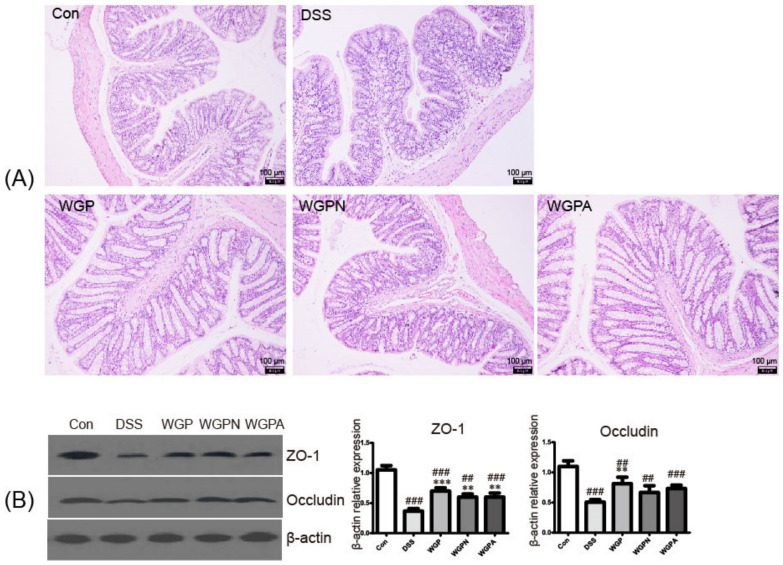
Changes of the colon structure and intestinal barrier: (**A**) histopathological observation; (**B**) intestinal barrier-related protein expression by Western blotting. The bars represent the mean ± SD, *n* = 3; ## *p* < 0.01, ### *p* < 0.001 compared with the con group; ** *p* < 0.01, *** *p* < 0.001 compared with the DSS group. Con, control group; DSS, colitis group; WGP, WGP treatment group; WGPN, WGPN treatment group; WGPA, WGPA treatment group.

The colon of the DSS model group exhibited significant tissue damage, such as the decrease in or disappearance of glands and goblet cells; a shallower crypt; disordered arrangement of intestinal villi; and severe epithelial injury and inflammatory cell infiltration in the mucosa and submucosa (Figure 2A). However, these damage signs were noticeably improved by ginseng polysaccharide fractions. The intestinal villi of the WGP and WGPA groups were longer, the crypts were deeper, the structures of the colonic glands were fuller and more complete, and the recovery effect was better than that of the WGPN group. Compared with the con group, the expression of the tight-junction protein ZO-1 and occludin were decreased in the DSS group (Figure 2B), suggesting the integrity and function of the colon–intestinal barrier were destroyed by DSS. Ginseng polysaccharides and their subfractions enhanced the expression of ZO-1 significantly; however, only WGP increased the expression of occludin significantly (Figure 2B). Compared with the con group, the expression of ZO-1 and occludin significantly decreased in the WGP, WGPN, and WGPA groups (Figure 2B). These results indicated that WGP and WGPA protected intestinal integrity and attenuated DSS-induced structure damage.

### 3.4. Ginseng Polysaccharide Fractions Decreased Intestinal Inflammation Level and Inhibited NF-κB Signaling Pathway

**Figure 3:**
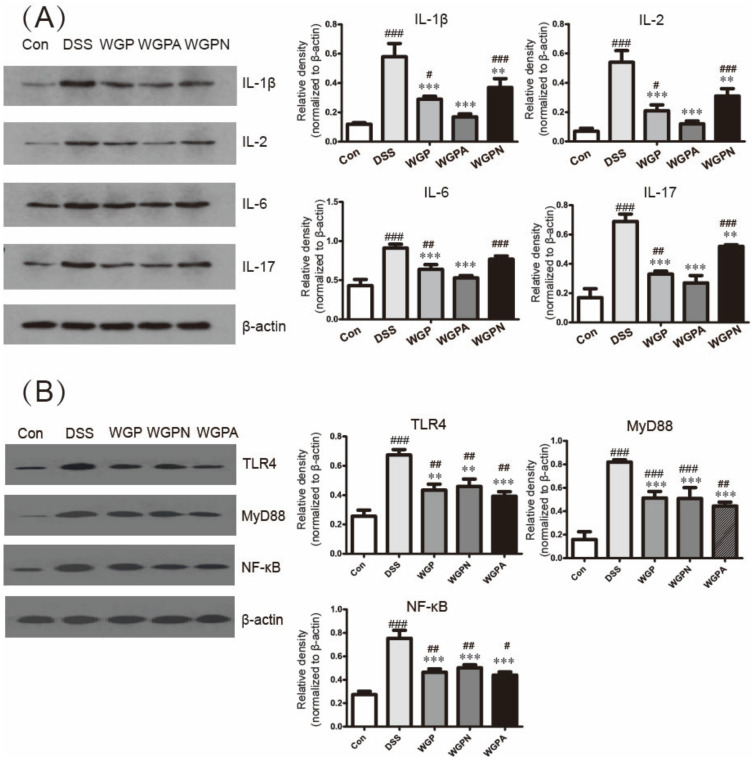
Changes of inflammatory-related protein expression in colon: (**A**) expression of cytokine using Western blotting and their relative densitometry; (**B**) expression of NF-κB pathway-related proteins using Western blotting and their relative densitometry. β-actin as loading control. The bars represent the mean ± SD, *n* = 3; # *p* < 0.05, ## *p* < 0.01, ### *p* < 0.001 compared with the con group; ** *p* < 0.01, *** *p* < 0.001 compared with the DSS group. Con, control group; DSS, colitis group; WGP, WGP treatment group; WGPN, WGPN treatment group; WGPA, WGPA treatment group.

The levels of inflammatory cytokines IL-1β, IL-2, IL-6, and IL-17 in the colonic mucosa were significantly increased in the DSS group compared with the con group (Figure 3A), which suggests an inflammation-causing effect of DSS in rats. WGP and its subfractions significantly improved the level of inflammation of the colon caused by DSS and downregulated the four inflammatory cytokines, among which the inflammatory cytokine levels in WGPN and WGPA were closer to those of the con group. Both WGPN and WGPA decreased the proinflammatory factors more effectively compared with WGP.

The NF-κB pathway-related protein level was detected using Western blotting (Figure 3B). DSS treatment increased the TLR4, MyD88, and NF-κB expression levels, which suggested the activation of the NF-κB pathway. After treatment with WGP, WGPN, and WGPA, the expression levels of the three proteins were downregulated, especially that of the WGPA group. It can be concluded that ginseng polysaccharides could improve the colitis inflammation via inhibition of the TLR4/MyD88/NF-κB pathway.

### 3.5. Ginseng Polysaccharide Fractions Adjusted the Diversity and Composition Changes of Gut Microbiota

**Figure 4:**
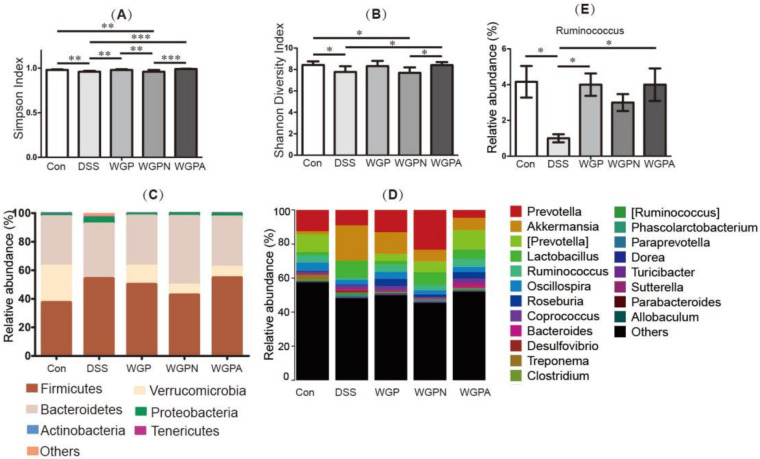
Diversity and composition analysis of the gut microbiota: (**A**) Simpson index; (**B**) Shannon index; (**C**) gut microbiota composition at the phylum level; (**D**) gut microbiota composition at the genus level; (**D**) relative abundance of Ruminococcus. The bars represent the mean ± SD (**A**,**B**,**E**) or the mean ± SEM (**C**,**D**), *n* = 8.* *p* < 0.05, ** *p* < 0.01, and *** *p* < 0.001.Con, control group; DSS, colitis group; WGP, WGP treatment group; WGPN, WGPN treatment group; WGPA, WGPA treatment group.

In the DSS group, the diversity of the gut microbiota was significantly decreased, as assessed using the Simpson index (Figure 4A) and the Shannon diversity index (Figure 4B). After the recovery treatment, the diversity indexes were obviously improved in the WGP and WGPA groups compared with the DSS group. However, the two α diversity indexes in the WGPN group did not show beneficial effects compared with the DSS group, although there were significant differences between the WGPN and the con groups.

Figure 4C showed the changes in the relative abundance of bacteria at the phylum level. The fecal microbiota of the five groups was mainly composed of Firmicutes, Verrucomicrobia, and Bacteroidetes. Compared with the con group, the relative abundance of Firmicutes and Bacteroidetes increased, while that of Verrucomicrobia decreased significantly in the DSS group, which suggested changes in the fecal microbiota composition under the DSS treatment. After treatment with the ginseng polysaccharide and its subfractions, the composition of the fecal microbiota also recovered. Compared with the DSS group, the relative abundance of Verrucomicrobia increased significantly in the three treatment groups, while that of Firmicutes and Bacteroidetes decreased in both WGP and WGPA groups, which was similar to the composition of the con group. The microbiota composition of the WGPN group was not similar to that of the DSS or con group compared with the WGP and WGPA groups and showed irregular variations after the recovery period, which is also consistent with the results obtained for the DAI, colon length, structure observation, and cytokine levels. Figure 4D,E shows the composition changes at the genus level. It can be concluded that the changes in the fecal microbiota focused on the relative abundance of *Prevotella*, *Akkermansia*, *Lactobacillu* and so on. Among all the changes, the relative abundance of *Ruminococcus* was significantly decreased in the DSS group compared with the con group. However, WGP and WGPA groups exhibited significant improvement in dysbiosis and an increase in the relative abundance of *Ruminococcus*. It suggested *Ruminococcus* might play an important role in the process of anti-colitis caused by ginseng polysaccharides.

### 3.6. Content of SCFAs in Feces

**Figure 5:**
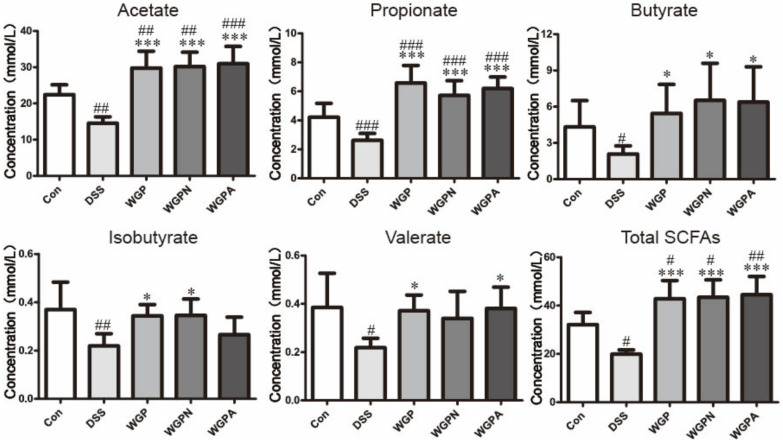
SCFA content in the feces of rats. The bars represent the mean ± SD, *n* = 8; # *p* < 0.05, ## *p* < 0.01, ### *p* < 0.001 compared with the con group; * *p* < 0.05, *** *p* < 0.001 compared with the DSS group. Con, control group; DSS, colitis group; WGP, WGP treatment group; WGPN, WGPN treatment group; WGPA, WGPA treatment group.

All types of SCFAs detected were significantly decreased in the DSS group compared with the con group (Figure 5). After treatment with WGP, WGAN, or WGPA, the content of SCFAs was significantly increased in feces compared with that observed in the DSS group. The contents of acetate, propionate, butyrate, and total SCFAs was enhanced most obviously in the three treatment groups (*p* < 0.001). However, WGPA increased the production of valerate and decreased that of isobutyrate, while WGPN yielded the opposite results compared with WGP. In general, the effects of WGP, WGPN, and WGPA on SCFA production were similar as all three polysaccharide fractions improved the levels of SCFAs in feces (Figure 5).

## 4. Discussion

In this study, compared with the DSS group, WGP and WGPA significantly improved the diarrhea status of rats, including a decrease in the DAI index, an increase in the colon length and colon weight, and a reduction in intestinal injury. It suggested that WGPA is the effective subfraction for the anti-colitis activity of WGP. Tight junctions (TJs) are the most apical intercellular complexes in epithelial cells and are composed of transmembrane barrier proteins (e.g., Claudins, occludin, and junctional adhesion molecules) and cytoplasmic scaffolding proteins (e.g., the ZO family, AF-6, and Cingulin) [23,24,25]. The downregulation of TJ proteins leads to an increase in the permeability of the intestinal epithelium, which might induce bacterial translocation, the risk of intestinal infection, and inflammation [26]. In this study, the expression of the occludin and ZO-1 proteins was significantly downregulated in the DSS group, which suggests that DSS destroys the mucosal layer and increases intestinal permeability. In contrast, WGP promoted the expression of ZO-1 and occludin in the colon significantly, suggesting that ginseng polysaccharides repair the intestinal epithelium after DSS damage, while the WGPN and WGPA treatments only promoted the levels of ZO-1. Therefore, regarding colon structure repair, WGP was more effective than WGPN and WGPA. The lamina propria T cells increase the expression of IL-1, IL-6, and IL-8 during the process of colitis [27,28]. The levels of inflammatory cytokines reflect the degree of inflammation of the intestinal mucosa. Here, we chose IL-1β, IL-2, IL-6, and IL-17 as representative cytokine factors with the aim of detecting changes in the levels of inflammatory molecules in the colon. It was shown that DSS aggravated the inflammation levels, while the three ginseng polysaccharides fractions significantly reduced the expression of these inflammatory cytokines in the colon. Concomitantly, the WGPN and WGPA sub-fractions exerted stronger effects on inflammation than did WGP.

The NF-κB pathway is a very important signal pathway in inflammation response. The occurrence of colitis was closely related with the NF-κB pathway [6]. The results of our study also identified the activation of the NF-κB pathway in colitis rats that was induced by DSS. Ginseng polysaccharides could significantly inhibit the activation of the NF-κB pathway via regulation of the TLR4 and MyD88 protein expression, especially WGPA treatment, further inhibiting the production of inflammation factors. Notably, we found that WGPN can also suppress this signaling pathway to a certain level, which was in accordance with the results of cytokine levels. These results suggested that although WGPN did not play a role in colitis treatment, it might have functions in other inflammatory diseases.

Dysbiosis of gut microbiota is an important feature of colitis. Here, we found that the Simpson and Shannon indexes of fecal microbiota decreased significantly in the DSS group compared with the con group, suggestive of the dysbiosis and the destruction of the structure of the gut microbiota. After treatment with WGP and WGPA, the diversity of fecal microbiota increased and became similar to that of the con group, which was indicative of recovery from dysbiosis. WGPN did not exert obvious effects on the diversity of fecal microbiota. These results indicate that the beneficial effects of WGP and WGPA on colitis might be associated with their abilities to adjust the diversity of the gut microbiota. We also found that WGP and WGPA recovered the composition of fecal microbiota at the phylum level. The microbiota changes afforded by WGP and WGPA that played a role in the DSS treatment were also observed at the genus level. The relative abundance of *Ruminococcus* significantly decreased in the DSS group compared with the con group; however, this abundance increased to normal levels after treatment with WGP or WGPA, while there was no significant change after treatment with WGPN. *Ruminococcus* is a common bacterium that produces SCFAs and has beneficial effects on hosts [29]. SCFAs are important metabolites in the intestinal microbial environment and are closely related to immune, anti-tumor, and anti-inflammatory activities [30]. Some plant polysaccharides that are not digested by the host are fermented by a series of anaerobic probiotics. Because of the different structures and sources of polysaccharides, different kinds of SCFAs are produced [31]. WGPA was rich in acidic pectin, which is often referred to as a prebiotic that ferments in the colon and produces beneficial metabolites, especially SCFAs. Since the gut microbiota adjusting ability, especially the increasing effects on Ruminococcus and the SCFA production, ginseng polysaccharide WGP and WGPA might be used as potential prebiotics, which help against diarrhea, gut microbiota dysbiosis, or other colonic-related diseases.

There have been some reports about the effect of ginseng on colitis, but these reports mostly focus on the effect of ginseng extract on colitis [32,33,34], but little attention is paid to the components of polysaccharides. It has been reported *Panax ginseng* polysaccharides (GPS) relieved the DAI, colon length shortening, and pathological changes in colonic tissue in mice with colitis, while adjusting oxidative level and inflammatory cytokines through inhibiting the JAK2/STAT1/NLRP3 pathway [15]. Our research further purified the ginseng polysaccharides into neutral polysaccharides (WGPN) and acidic polysaccharides (WGPA) and identified the effective subfraction while exploring the mechanism from the perspective of the NF-KB-signaling pathway. WGPN is a starch-like glucan that belongs to the group of neutral polysaccharides. In contrast, WGPA is an acidic pectin that is rich in type I rhamnogalacturonan and homogalacturonan [17]. The different structural features of these sub-fractions determine their different activities. Of the two purified fractions, WGPA showed more obvious beneficial effects on colitis compared with WGPN, not only regarding the normal status, but also regarding gut microbiota diversity and composition. We can conclude that the acidic region, such as galacturonic acid-rich parts in the WGPA, might play an important role in the anti-colitis activity. Although such results might need more research to identify, they could provide some information for us to search for natural products with anti-colitis activity.

Although some positive results have been obtained on the effects and mechanism of ginseng polysaccharides on colitis in this research, it should be noted that there are still some deficiencies and limitations. For example, in terms of mechanism research, the details of the signaling pathway were not fully discussed in this research. In the following work, we will focus more on these aspects and further study the therapeutic effect of ginseng polysaccharides on colitis and its specific mechanism to provide a data basis for conquering colitis.

## 5. Conclusion

In conclusion, the ginseng polysaccharide extract, WGP, improved the symptoms of DSS-induced colitis in rats, with the acidic pectin fraction, WGPA, playing the main role in this activity. WGP and WGPA improved the symptoms of colitis by reducing the expression level of inflammatory cytokines in the colon; maintaining the integrity of the intestinal barrier via the upregulation of tight junction-related proteins; regulating the diversity and composition of gut microbiota; increasing the relative abundance of *Ruminococcus*; increasing the production of SCFAs; and inhibiting the TLR4/MyD88/NF-κB signaling pathway. The results of this study may provide the basic data for improving the effects of natural polysaccharides on colitis and promote the applications of *Panax ginseng* and its active components.

## Figures and Tables

**Table 1:** DAI score criterion.

Scores	Weight Loss/(%)	Stool Consistency	Stool Occult Blood
0	−	Normal stool	Blood negative (−)
1	1–5	Soft stool	Blood positive (±)
2	5–10	Loose stool	Stool with slight blood (+)
3	10–15	Unformed stool	Stool with blood (++)
4	>15	Watery diarrhea	Stool with severe blood (>++)

**Table 2:** Yield and physiochemical features of collected fractions.

Fraction	Yield (%)	Total Carbohydrate Contents (%)	Uronic Acid Contents (%)	Protein Contents (%)	Monosaccharide Composition (%)				
					Glc	Gal	Ara	GalA	Rha
WGP	10.1 ^1^	89.2	22.9	0.6	42.6	15.1	13.9	25.7	2.7
WGPN	61.8 ^2^	92.8	0	0.3	95.3	3.3	1.4	---	---
WGPA	26.5 ^2^	92.1	38.5	0.2	13.6	18.0	15.4	44.2	3.8

^1^ Yield in relation to the dried ginseng roots; ^2^ Yield in relation to the weight of WGP applied into column.

## Data Availability

Not applicable.
